# University commitments to a sustainable food system and lessons for the global south

**DOI:** 10.1017/S1368980025101572

**Published:** 2025-12-23

**Authors:** Jeff Clyde Corpuz

**Affiliations:** Department of Theology and Religious Education, College of Liberal Arts, De La Salle University, 2401 Taft Avenue, Manila 1004, Philippines

**Keywords:** Food, University, Sustainability, Public health

Dear Editor,

Blennerhassett et al. provide the most comprehensive review to date of food sustainability (FS) policies across UK universities, examining 163 institutions and finding that only 51·5 % had a publicly available policy, with commitments most frequently addressing communication, food waste and certification standards, but with far fewer addressing research and innovation (17·9 %)^(1)^. Their content analysis underscores both promise and limitation: universities are increasingly framing FS within operational domains but remain hesitant to integrate research, pedagogy and structural reform. For the Global South, where higher education institutions (HEIs) face different constraints and opportunities, these findings are instructive. Several key implications emerge.

UK institutions show progress in making policies public, but measurable targets remain scarce, especially regarding meat reduction and plant-based transitions^(1)^. In low- and middle-income countries (LMICs), where informal food systems dominate, transparency is often weaker still. HEIs in the Global South must prioritise publishing policies with clear benchmarks, aligning with both national climate targets and local food security needs. This requires not only adopting sustainability frameworks but also adapting them to contexts of poverty, food insecurity, and agricultural dependency.

Blennerhassett et al. highlight the striking absence of research and innovation within FS policies, despite universities’ role as knowledge producers^(1)^. For HEIs in Africa, Asia, and Latin America, this gap is a crucial opportunity^(2)^. Agricultural faculties and public health programmes can link sustainable procurement with student-led research, for example, testing agroecological practices in campus farms or conducting behavioural interventions in canteens. Embedding FS into curricula can prepare graduates to reform food systems beyond campus walls.

The authors note a disjunction between universities’ research agendas and their catering practices, a tension termed the cognitive–practice gap^(1)^. In the Global South, this risk is compounded by resource scarcity and donor-driven agendas. Closing this gap requires aligning institutional procurement with the food sovereignty movements active across many LMICs. HEIs can model dietary shifts by supporting indigenous crops, reducing ultra-processed foods, and valorising local cuisines that are both culturally relevant and environmentally sustainable.

The UK data show that most policies neglect broader social sustainability dimensions, such as labour rights or farmer livelihoods. In the Global South, such omissions would be fatal. Universities must explicitly commit to fair procurement from smallholder farmers, women’s cooperatives, and community enterprises. This supports local economies and ensures FS policies do not reinforce existing inequalities in global food chains.

The UK experience reveals incremental but fragmented commitments. For HEIs in the Global South, the lesson is twofold: adopt the strengths of multidimensional FS commitments but avoid the weaknesses of vague, non-measurable goals. Universities must act as both exemplars and incubators of food system transformation, integrating operational policy, student engagement, and community outreach.^(2)^ If pursued with clarity and equity, higher education in the Global South can reshape food environments in ways that simultaneously reduce climate risks, strengthen local economies, and improve health outcomes.

## Data Availability

The author has nothing to report.
